# The Actions and Mechanisms of P2X7R and p38 MAPK Activation in Mediating Bortezomib-Induced Neuropathic Pain

**DOI:** 10.1155/2020/8143754

**Published:** 2020-07-14

**Authors:** Yan Guo, Xiaobo Xu, Jingyi Huang, Zhen Wang, Zhenzhong Li, Zhen Liu

**Affiliations:** ^1^Department of Anatomy and Neurobiology, Shandong University School of Basic Medical Sciences, Jinan 250012, China; ^2^Department of Human Biology and Cell & Systems Biology, University of Toronto, Toronto, Ontario, Canada M5S 3J6

## Abstract

The proteasome inhibitor bortezomib (BTZ) is a potent first-line anticancer drug for multiple myeloma; nonetheless, it induced peripheral neuropathy. It has been suggested that many cytokines may play a role in mediating neuropathic pain, but the underlying molecular mechanism is not fully understood. Recent studies have shown that neuropathic pain is closely related to the purinergic ligand-gated ion channel 7 receptor (P2X7R), one of the P2X receptors, which is richly expressed in glial cells. P2X7-p38 pathway is correlated with microglia- and satellite glial cell- (SGC-) mediated neuropathic pain. However, the association of P2X7R and p38MAPK in mediating BTZ-induced neuropathic pain remains unclear. In this study, the relationship between P2X7R activation and p38 phosphorylation in the dorsal root ganglion (DRG) and spinal dorsal horn (SDH) in the development and maintenance of BTZ-induced neuropathic pain was elucidated. The results showed that BTZ increased mechanical thresholds in rats, accompanied with upregulation of P2X7R expression and p38MAPK phosphorylation, indicating that P2X7R and p38MAPK are key molecules in the development and maintenance of BTZ-induced neuropathic pain. Inhibiting p38MAPK phosphorylation with SB203580 resulted in downregulation of P2X7R expression levels. Inhibition of P2X7R with Brilliant Blue G (BBG) reversed neuropathic pain might decrease through the expression of tumor necrosis factor-*α* (TNF-*α*), interleukin-1*β* (IL-1*β*), and IL-6 via inhibiting p38MAPK phosphorylation. The P2X7R/p38MAPK signaling pathway in SGCs of DRG and microglia of SDH might be a potential pharmacological target behind this mechanism as an opportunity to relieve BTZ-induced neuropathic pain.

## 1. Introduction

Proteasome inhibitors used for treating cancers have been identified to result in a significant higher risk for inducing sensory peripheral neuropathy [1]. Bortezomib (BTZ), a proteasome inhibitor, is a commonly used antitumor drug which has highly effective results on relieving cancer symptoms [2, 3]. Bortezomib-induced peripheral neuropathy (BIPN) is a major and debilitating side effect with persistent exacerbation of neuropathic pain during anticancer therapy, which causes drug dose limitation or application discontinuation and worsens cancer prognosis [4]. With no viable treatment options, it is crucial to mitigate BIPN when using BTZ as an anticancer therapy.

Neuroinflammation is one of the major contributors to the initiation of BIPN; as most recently, it has been shown that persistent neuroinflammation and progressive structural damage of the dorsal root ganglion (DRG) in addition to neuroinflammation-related spinal cord sensitization were associated with development of BIPN in mice [5]. Studies have confirmed that proinflammatory cytokines like tumor necrosis factor-*α* (TNF-*α*), interleukin-1*β* (IL-1*β*), and IL-6 can activate p38MAPK, while activated p38 can also lead to the release of inflammatory mediators [6]. It leads to the induction and expansion of neuropathic pain status. p38MAPK phosphorylation in the central nervous system [7, 8] and peripheral nervous system [9–11] is closely correlated with neuroinflammation and neuropathic pain.

Primary DRG neurons transmit sensory information about pain, in which ATP-dependent P2X receptors are richly expressed [11]. Studies have shown that P2X family receptors expressed in DRG satellite glial cells (SGCs) play an important role in regulating neuropathic pain [12, 13]. P2X7 receptor (P2X7R) is one of the P2X receptors involved in nociceptive signaling with some obvious characteristics like requiring sufficient concentration of ATP activating and stimulating for a long time to participate in the potentiation on its cation channel function [14]. This pathway may be a potential mechanism of central and peripheral nervous system disorders as a result of neuropathic pain. P2X7R is located in microglia of the central nervous system and in SGCs of the DRG [15, 16]. Activation of P2X7R in microglia of the spinal cord and in SGCs of DRG contributes to inflammatory pain [9, 17].

The role of P2X7R in pain has been demonstrated in animal models of neuropathic and inflammatory pain [18, 19]. P2X7R gene-deficient mice lack pain-related behavioral hypersensitivity caused by partial nerve ligation [20]. Reducing the expression of P2X7R in the DRG could relieve diabetic neuropathic pain in rats [21]. In addition, some P2X7R-specific antagonists have been shown to be effective in alleviating neuropathic pain and nerve injury animal models [22, 23]. Furthermore, the P2X7R-p38 pathway is correlated with microglia-mediated neuropathic pain [24] and SGC-mediated neuropathic pain [25, 26], suggesting a close relationship between P2X7 activation and p38 phosphorylation in contributing to the development and maintenance of neuropathic pain.

Although by increasing surveillance and earlier dose adjustment of the proteasome inhibitor could minimize the risk of sensory peripheral neuropathy in clinical trials [1] and drugs for preventing BIPN [27], neurotoxic mechanisms leading to BIPN still need to be further investigated in order to prevent or treat this debilitating adverse effect. At the moment, the high prevalence of BIPN constitutes a major problem for these patients who received BTZ therapy; thus, a better understanding of the underlying mechanisms of BIPN is urgently needed for developing available preventive and therapeutic measurements [28]. Although a multitude of molecular and cellular mechanisms are raised to explain the BTZ-induced painful peripheral neuropathy in animal models [5, 29–36], the association of the P2X7R and p38 signaling pathways in mediating BTZ-induced neuropathic pain is still unclear. In the present study, a BIPN rat model was established and used to elucidate the underlying novel pathogenesis and mechanisms involved in P2X7R activation and p38 phosphorylation in specific cell types of both DRG and SDH tissues. These findings will help to develop new therapeutic strategies for preventing or mitigating BIPN by interfering P2X7R activation and p38 phosphorylation states in these distinct cell types. The mechanism and its potential therapeutic strategies showed promise to shed light on alleviating neuropathic pain in cancer treatment.

## 2. Materials and Methods

### 2.1. Ethics Statement

The Wistar rats were from Shandong University Experimental Animal Center. All animal experimental procedures were in accordance with the 8th Edition of the *Guide for the Care and Use of Laboratory Animals* (https://grants.nih.gov/grants/olaw/Guide-for-the-care-and-use-of-laboratory-animals.pdf) published by the National Academy of Sciences, The National Academies Press, Washington DC, United States of America. These experimental procedures were approved by the Animal Experimentation Ethical Committee of Shandong University (Document No. ECSBMSSDU-2018-2-007).

### 2.2. Animal Groups and Experimental Design

A total of 40 male Wistar rats were used in this study. Twenty rats were randomly divided into the following 4 groups (each group consisted of 5 rats). (1) Control group: rats received peritoneal injection (i.p.) of 0.9% NaCl at the equivalent volume the other groups. (2) Brilliant Blue G (BBG) group: rats received BBG (50 mg/kg body weight, i.p.) at a concentration of 10 mg/ml. BBG is a P2X7R inhibitor which was used in this experiment aimed to clarify whether this inhibitor alone affects the experimental results. (3) BTZ group: rats received BTZ (0.2 mg/kg body weight, i.p.) at a concentration of 0.5 mg/ml for 5 successive days. (4) BTZ+BBG group: rats received BBG (50 mg/kg body weight, i.p.) 30 min before BTZ (0.2 mg/kg body weight, i.p.) administration with the corresponding concentration. Another 20 rats were randomly divided into the following 4 groups (each group of 5 rats). (1) Control group: rats received intrathecal injection (i.t.) of 0.9% NaCl at the equivalent volume the other groups. (2) SB203580 group: rats received SB203580 (10 *μ*l, i.t.) at a concentration of 10 *μ*g/10 *μ*l. SB203580 is a selective p38 inhibitor which was used in this experiment aimed to clarify whether this inhibitor alone affects the experimental results. (3) BTZ group: rats received BTZ (0.2 mg/kg body weight, i.p.) at a concentration of 0.5 mg/ml for 5 successive days. (4) BTZ+SB203580 group: rats received SB203580 (10 *μ*l, i.t.) 30 min before BTZ (0.2 mg/kg body weight, i.p.) administration with the corresponding concentration.

### 2.3. Drug Administration

BTZ (0.2 mg/kg, 0.5 mg/ml) and P2X7R antagonist BBG (50 mg/kg, 10 mg/ml) were administered via peritoneal injection (i.p.) according to the above experimental design. p38MAPK inhibitor SB203580 (10 *μ*g in 10 *μ*l) was administered by intrathecal injection (i.t.) through a PE-10 intrathecal catheter implanted at the L5 spinal segmental level. Animals receiving different reagents were anesthetized with 1.5% sodium pentobarbital (2 ml/kg body weight). A sterile PE-10 tube filled with saline was inserted through the L5/L6 intervertebral space. The right position of catheter placement was verified by observing transient hind paw paralysis induced by intrathecal injection of lidocaine (2%, 10 *μ*l) at the end of the experiments.

### 2.4. Mechanical Allodynia Behavior Test with von Frey Filament

The pain thresholds of mechanical stimulation were measured with von Frey filaments before (day 0) and after (days 2, 4, 6, 8, and 10) BTZ treatment. The protocol of the mechanical allodynia behavior test was similar to our previous reports [37, 38]. Thirty minutes of accommodation time before the behavior test for each rat was needed. The average score of the hind paw threshold was calculated from 3 successive tests with 5 minutes interval. All the behavior tests were carried out in a blind manner.

### 2.5. Quantitative Real-Time RT-PCR for Detecting mRNA Levels

After treatment with different reagents and the last mechanical simulation behavior test finished, fresh L4-6 DRG and the corresponding segment SDH tissue were removed from each animal under anesthesia. Total RNA of DRG and SDH tissue was extracted with TRIzol. cDNA was synthesized with a cDNA synthesis kit (Thermo Scientific). Quantitative PCR was performed with SYBR Green master mixes (Cwbiotech), and amplification was performed with synthetic oligonucleotide primers. The PCR reaction was performed at 55°C for 2 minutes, 95°C for 10 minutes, followed by 40 cycles at 94°C for 5 seconds, 58°C for 30 seconds, and 72°C for 45 seconds and the final results carried out by using the 2^-*ΔΔ*Ct^ method [39]. The primer sequences are shown in Table 1.

### 2.6. Western Blot for Examining Protein Levels

Upon L4-6 DRG and the corresponding segment SDH tissue removal, the DRG and SDH tissue was prepared for Western blot assay for detecting the variation of the targeting proteins. DRG and SDH tissue was lysed in RIPA buffer (Beyotime Biotechnology) containing protease and phosphatase inhibitors (Roche) on ice for 20 minutes and removed to a refrigerated centrifuge at 12000 rpm for 15 minutes to collect supernatant. The protein samples (50 *μ*g) were loaded into each lane, and total protein was separated in SDS-PAGE gel. Nitrocellulose membrane was used to transfer and incubated overnight at 4°C with each primary antibody. The corresponding secondary antibody was incubated at room temperature for 2 hours. The primary antibodies and secondary antibodies are shown in Table 2. The immunoreactive images were analyzed quantitatively using ImageJ software.

### 2.7. Immunofluorescence Labeling

After treatment with different reagents and the last mechanical simulation behavior test finished, the animals were anaesthetized and perfused with cold (4°C) 4% paraformaldehyde (pH 7.4). L4-6 DRG and the corresponding segment SDH tissue were removed from each animal. DRG and SDH tissue slide of 15 *μ*m thickness was made by monitoring with a freezing microtome (AS620 Cryotome, UK). Cell permeabilization of DRG and SDH tissue was incubated with 0.5% Triton X-100 for 60 min at room temperature. Nonspecific binding site was blocked with 10% normal goat serum in 1× PBS for 1 hour. The slide was incubated with each primary antibody overnight at 4°C, followed by incubation with the corresponding secondary antibody at 37°C. The primary antibodies and secondary antibodies used in immunofluorescence labeling are shown in Table 3. The slides were rinsed in 1× PBS for 3 times between each step. After finishing the fluorescence staining, the slides were mounted with antifade mounting medium (Abcam) and were observed with a fluorescence microscope (Olympus BX63). The images taken by a microscope were analyzed with ImageJ software or Image-Pro Plus (version 6.0) according to the cell types or distinct markers.

### 2.8. Statistical Analysis

*Mean* ± *SEM* was used for reporting the quantitative data obtained in this study. SPSS (version 19.0) was used for the statistical analysis. The nonparametric test was used for analyzing abnormal distributed data. The normal distributed data were assessed with one-way analysis of variance followed by the Student-Newman-Keuls test (homogeneity of variance) or Dunnett's T3 test (heterogeneity of variance). *P* value < 0.05 was taken as significant.

## 3. Results

### 3.1. The Mechanical Threshold Measurement before and after BTZ Treatment

**Figure 1:**
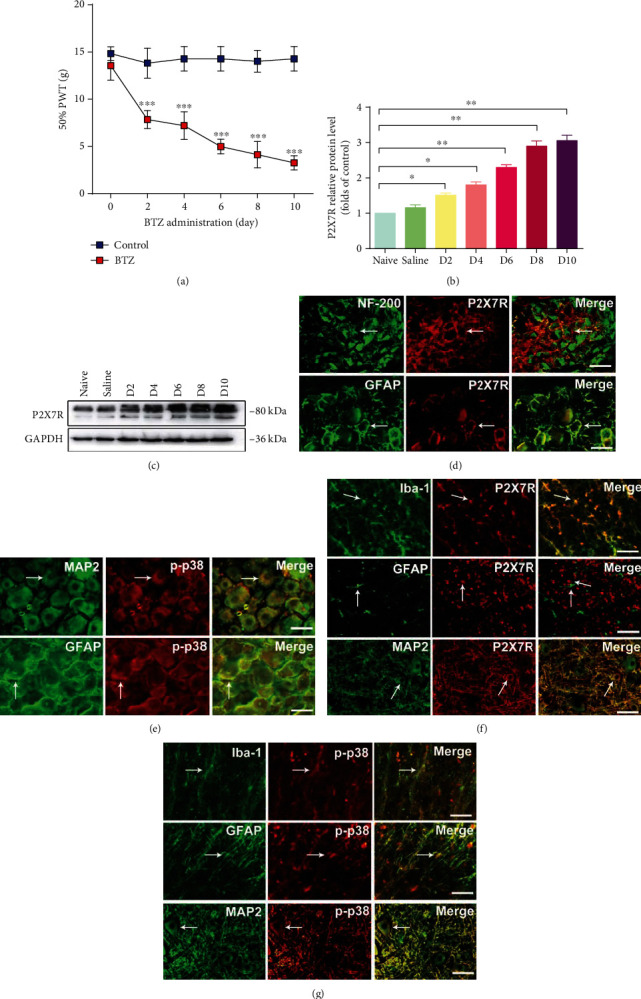
Mechanical threshold and P2X7R and p-p38 expression. (a) Mechanical threshold after BTZ injection. (b, c) Western blot for P2X7R expression after BTZ treatment. (d) Immunofluorescence location of P2X7R in DRG. The arrows indicate the typical single- or double-labeled DRG neurons and satellite cells. P2X7R is not expressed in NF-200-positive neurons. P2X7R is expressed in GFAP-labeled satellite glial cells (SGCs). (e) Immunofluorescence location of p-p38 in DRG. The arrows indicate the typical single- or double-labeled DRG neurons and satellite cells. p-p38 is expressed in both MAP2-labeled neurons and GFAP-labeled SGCs. (f) Immunofluorescence location of P2X7R in SDH. The arrows indicate the typical single-labeled and double-labeled cells in SDH. P2X7R is expressed mainly in Iba-1-labeled microglial cells rather than in GFAP-labeled astrocytes and MAP2-labeled neurons. (g) Immunofluorescence location of p-p38 in SDH. The arrows indicate the typical single-labeled and double-labeled cells in SDH. p-p38 is expressed mainly in Iba-1-labeled microglial cells. Scale bar = 50 *μ*m. Mean ± SEM (*n* = 5). ^∗^*P* < 0.05; ^∗∗^*P* < 0.01; ^∗∗∗^*P* < 0.001.

To clarify BTZ injection on mechanical allodynia behavior in rats, the mechanical thresholds of the bilateral plantar surface were measured by using the von Frey filament test before (0 day) and after 2, 4, 6, 8, and 10 days from the first BTZ injection. The mechanical threshold was decreased to a much lower point after 10 days from the first BTZ injection (Figure 1(a)), which suggested that BTZ induced an obvious mechanical allodynia at this time point. Thus, BTZ-induced peripheral neuropathy with allodynia rat model was successfully established. Animal model was used to perform the experiment as designed in Materials and Methods.

### 3.2. Alterations of P2X7R Expression after BTZ Treatment

To investigate the successive expression pattern of P2X7R in DRG after BTZ administration, P2X7R expression in DRG tissue from rats after 2, 4, 6, 8, and 10 days from the first BTZ injection was analyzed with Western blot. P2X7R protein elevation was observed from 2 days after BTZ injection and gradually upregulated to the highest levels between 8 and 10 days after BTZ injection (Figures 1(b) and 1(c)). This result suggested that P2X7R protein expression was significantly increased 8-10 days after the first BTZ injection. Hence, in this study, the observation time of BTZ toxic experiment was determined as 10 days after the first BTZ injection in all the following experiments.

### 3.3. Determination of P2X7R and p-p38 in Specific Cell Types in DRG and SDH

To determine what cell types express P2X7R and p-p38 in DRG and SDH, we carried out a series of experiments by using DRG and SDH tissue sections with double immunofluorescence labeling technique to approach these questions. In DRG tissue sections, P2X7R and GFAP (a satellite glial cell marker) double fluorescence labeling showed that P2X7R was coexpressed with GFAP, whereas P2X7R and NF-200 (a neuronal marker) double fluorescence labeling showed that P2X7R was not coexpressed with NF-200 (Figure 1(d)). As a result, P2X7R is expressed in SGCs rather than in neurons of the DRG. In DRG tissue sections, fluorescence labeling showed that p-p38 is expressed both in DRG neurons and SGCs (Figure 1(e)). In SDH tissue sections, P2X7R, and Iba-1 (microglia marker), double fluorescence labeling showed that P2X7R was coexpressed with Iba-1, whereas P2X7R with GFAP (an astrocyte marker) and MAP2 (a neuronal marker) double fluorescence labeling showed that P2X7R was not coexpressed with GFAP or MAP2 (Figure 1(f)). These results indicated that P2X7R is expressed in microglial cells rather than in astrocytes and neurons. On the other hand, p-p38 with specific cell markers double fluorescence labeling showed that p-p38 mainly expressed in microglial cells in SDH (Figure 1(g)). The location of P2X7R and p-p38 expression in specific cell types is particularly important for our coming experiments in this study. In the following experiments, we will focus on the specific cell types which expressed P2X7R or p-p38 in both DRG and SDH to clarify the mechanisms of BTZ-induced neuropathy mediated by P2X7R and/or p-p38.

### 3.4. p38 Phosphorylation in DRG and SDH after Inhibition of P2X7R

To clarify p38 phosphorylation in DRG and SDH after BTZ treatment with P2X7R inhibition, BTZ-treated rats received P2X7R inhibitor BBG. After 10 days of the first BTZ injection, DRG and SDH tissue was removed from each animal. The p38 mRNA, p-p38 protein levels, and p-p38 expression in situ in DRG or SDH tissue were analyzed with quantitative real-time PCR, Western blot, and fluorescence labeling, respectively.

**Figure 2:**
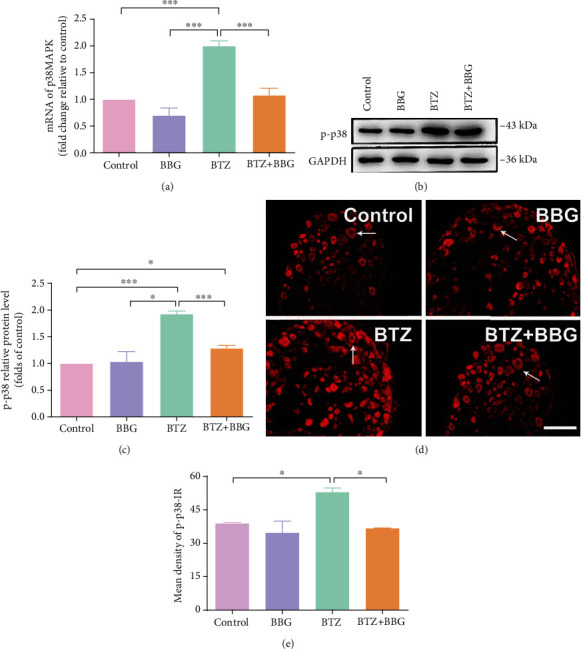
p38 mRNA expression and p38 phosphorylation in DRG after inhibition of P2X7R with BBG. (a) p38 mRNA levels. (b) p-p38 protein immunoblotting bands. (c) p-p38 protein levels. (d) p-p38 immunofluorescence labeling. The arrows show the typical p-p38 single-labeled DRG cells. (e) p-p38 fluorescence density. Scale bar = 50 *μ*m. Mean ± SEM (*n* = 5). ^∗^*P* < 0.05; ^∗∗∗^*P* < 0.001.

In DRG, BTZ treatment triggered a significant higher upregulation of p38 mRNA levels (Figure 2(a)) and 2p-p38 protein levels (Figures 2(b) and 2(c)). The p-p38 fluorescence intensity also increased significantly in DRG neurons (Figures 2(d) and 2(e)). To determine the role of p38 in the P2X7R regulated neuropathy pathway, the p-p38 expression was determined in the presence of the P2X7R antagonist, Brilliant Blue G (BBG). Inhibition of P2X7R with BBG in BTZ-treated rats significantly decreased p38 mRNA and p-p38 expression in DRG neurons. Application of BBG alone in naïve rats did not affect p38 mRNA and p-p38 expression in DRG neurons. These results suggested that activation of P2X7R in SGCs is closely related to p38 phosphorylation in DRG neurons from BTZ-induced neuropathic rats. BBG inhibited the level of p-p38, suggesting that p38 activity is regulated by P2X7Rs. This might be one of the novel mechanisms clarified in this experiment for the first time in BTZ-induced painful peripheral neuropathy.

**Figure 3:**
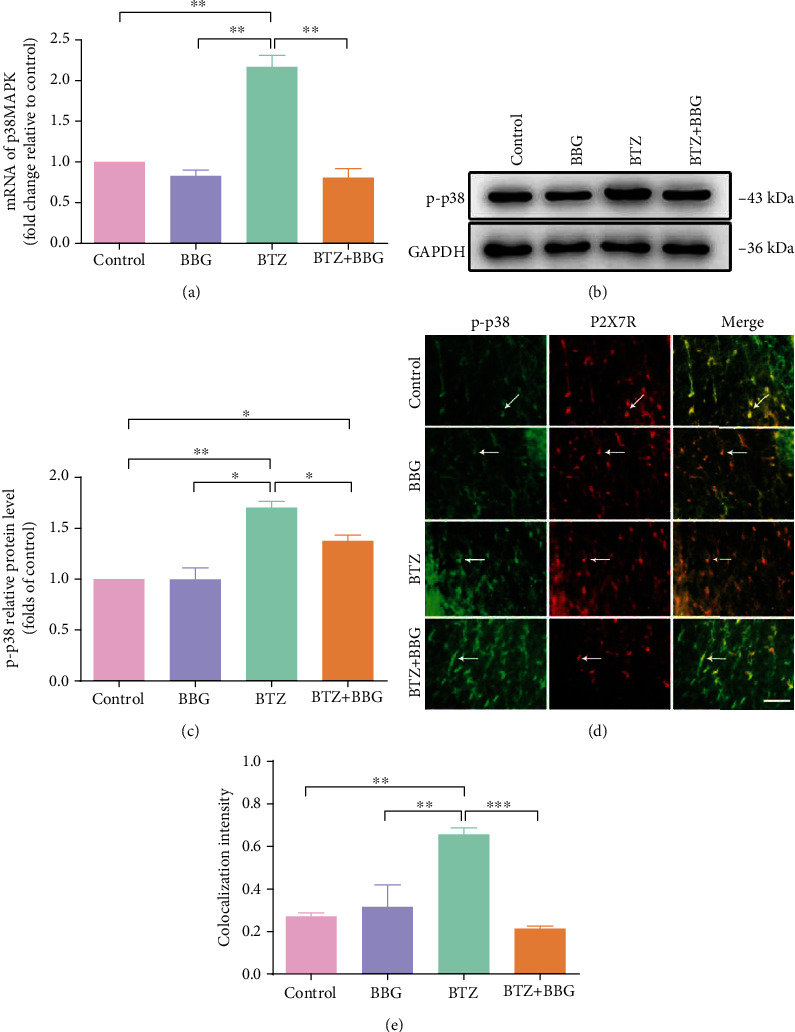
p38 mRNA expression and p38 phosphorylation in SDH after inhibition of P2X7R with BBG. (a) p38 mRNA levels. (b) p-p38 protein immunoblotting bands. (c) p-p38 protein levels. (d) P2X7R and p-p38 coexpression fluorescence labeling. The arrows indicate the typical single-labeled and double-labeled SDH microglia. (e) P2X7R and p-p38 coexpression fluorescence density. Scale bar = 50 *μ*m. Mean ± SEM (*n* = 5). ^∗^*P* < 0.05; ^∗∗^*P* < 0.01; ^∗∗∗^*P* < 0.001.

In SDH, p38 mRNA levels (Figure 3(a)) and 3p-p38 protein levels (Figures 3(b) and 3(c)) were significantly elevated in the BTZ treatment. The fluorescence intensity of p-p38 and P2X7R colocalization in microglia also increased significantly in SDH tissue (Figures 3(d) and 3(e)).

Inhibition of P2X7R with a P2X7R inhibitor BBG in BTZ-treated rats significantly decreased p38 mRNA and p-p38 expression in SDH tissue. Administration of BBG alone in naïve rats did not affect p38 mRNA and p-p38 expression in SDH tissue. These results implied that the activation of P2X7R in microglia of SDH is closely related to p38 phosphorylation in microglia of SDH. This might be another novel mechanism clarified in this experiment for the first time in BTZ-induced painful peripheral neuropathy.

### 3.5. IL-1*β*, IL-6, and TNF-*α* mRNA Expression in DRG and SDH after Inhibition of P2X7R

**Figure 4:**
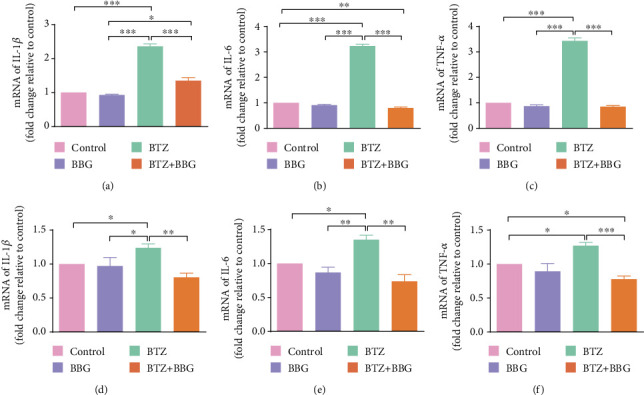
IL-1*β*, IL-6, and TNF-*α* mRNA expression in DRG and SDH after inhibition of P2X7R. (a) DRG IL-1*β* mRNA. (b) DRG IL-6 mRNA. (c) DRG TNF-*α* mRNA. (d) SDH IL-1*β* mRNA. (e) SDH IL-6 mRNA. (f) SDH TNF-*α* mRNA. Mean ± SEM (*n* = 5). ^∗^*P* < 0.05; ^∗∗^*P* < 0.01; ^∗∗∗^*P* < 0.001.

It has been shown that inflammatory cytokines such as IL-1*β*, IL-6, and TNF-*α* are involved in the progression of BTZ-induced peripheral neuropathy. Whether expression of these inflammatory cytokines is related to P2X7R activation in BTZ-induced peripheral neuropathy is yet to be clarified. In this study, IL-1*β*, IL-6, and TNF-*α* mRNA expression levels were determined by using a quantitative real-time PCR technique in DRG and SDH from BTZ-treated rats with or without inhibition of P2X7R. The results showed that the elevated IL-1*β*, IL-6, and TNF-*α* mRNA levels in both DRG and SDH tissues induced by BTZ treatment could be significantly blocked by administration of P2X7R inhibitor BBG (Figure 4). These results implied that BTZ-triggered inflammatory cytokine upregulation in both DRG and SDH tissues could be inhibited by P2X7R inhibition, which might relate to the alleviation of BTZ-induced peripheral neuropathy.

### 3.6. P2X7R Expression in DRG and SDH after Inhibition of p38

To determine whether P2X7R is expressed in DRG and SDH after BTZ treatment with p38 inhibition, the p38 selective inhibitor SB203580 was injected into BTZ-treated rats. The P2X7R expression in DRG or SDH tissue was analyzed with quantitative real-time PCR, Western blot, and fluorescence labeling, respectively.

**Figure 5:**
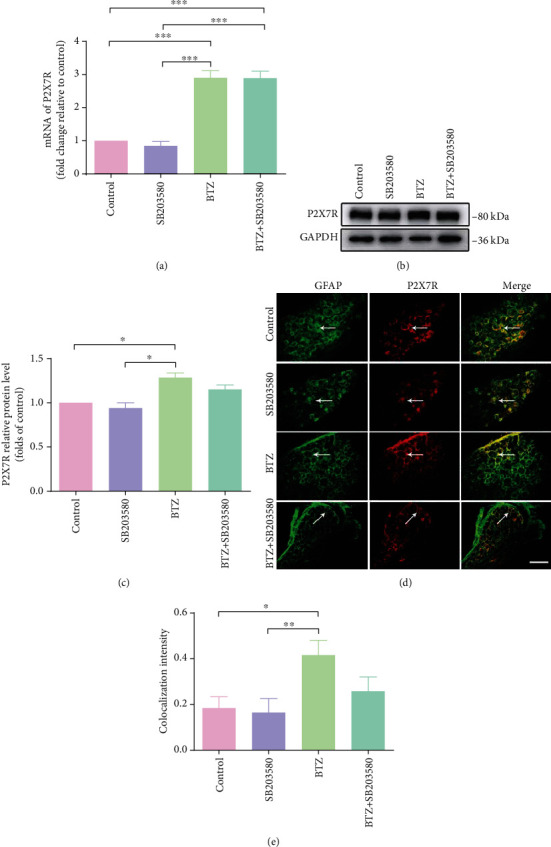
P2X7R mRNA and protein expression in DRG after inhibition of p38 phosphorylation. (a) P2X7R mRNA levels. (b) P2X7R protein immunoblotting bands. (c) P2X7R protein levels. (d) P2X7R and GFAP coexpression fluorescence labeling for SGCs. The arrows indicate the typical single-labeled and double-labeled DRG satellite cells. (e) P2X7R and GFAP coexpression fluorescence density. Scale bar = 50 *μ*m. Mean ± SEM (*n* = 5). ^∗^*P* < 0.05; ^∗∗^*P* < 0.01; ^∗∗∗^*P* < 0.001.

In DRG, BTZ treatment triggered a significant higher upregulation of P2X7R mRNA levels (Figure 5(a)) and P2X7R protein levels (Figures 5(b) and 5(c)). The P2X7R fluorescence intensity also increased significantly in SGCs of DRG (Figures 5(d) and 5(e)). Inhibition of p38 phosphorylation with the p38 selective inhibitor SB203580 did not significantly affect P2X7R expression in both naïve and BTZ-treated rats. These results suggest that DRG SGCs P2X7Rs play a major role in the regulation of p38 expression. These results also suggested that BTZ-induced P2X7R expression in DRG was not related to p38 phosphorylation.

**Figure 6:**
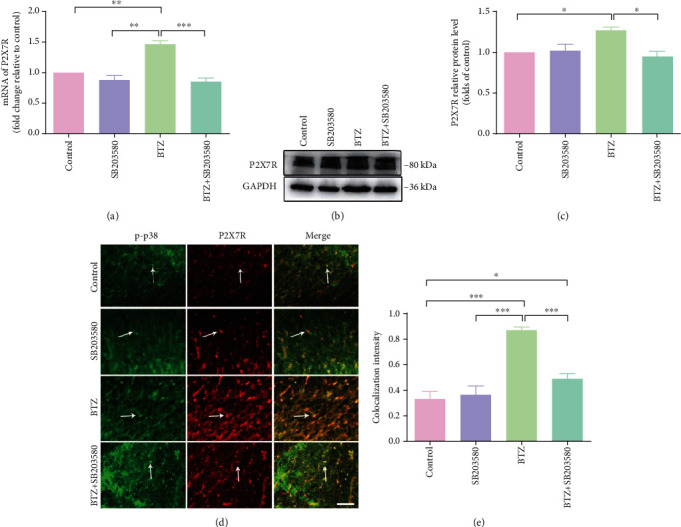
P2X7R mRNA and protein expression in SDH after inhibition of p38 phosphorylation. (a) P2X7R mRNA levels. (b) P2X7R protein immunoblotting bands. (c) P2X7R protein levels. (d) P2X7R and p-p38 coexpression fluorescence labeling. The arrows indicate the typical single-labeled and double-labeled SDH microglia. (e) P2X7R and p-p38 colocalization fluorescence density. Scale bar = 50 *μ*m. Mean ± SEM (*n* = 5). ^∗^*P* < 0.05; ^∗∗^*P* < 0.01; ^∗∗∗^*P* < 0.001.

In SDH, BTZ treatment induced a significant upregulation of P2X7R mRNA levels (Figure 6(a)) and P2X7R protein levels (Figures 6(b) and 6(c)).Western blotting analyses showed that inhibition of p38 phosphorylation with the p38 selective inhibitor SB203580 in BTZ-treated rats significantly decreased P2X7R expression in SDH tissue. However, administration of SB203580 in naïve rats did not affect P2X7R expression in SDH tissue. These results are further supported by the immunohistochemical study showing that the fluorescence intensity of P2X7R and p-p38 colocalization in microglial cells also changed significantly in SDH tissue (Figures 6(d) and 6(e)). These observations implied that P2X7R expression is closely related to p38 phosphorylation in microglia of SDH, suggesting the phosphorylation of p38 promotes P2X7Rs activity to exert control on neuropathic pain at SDH level. From these observations, we conclude that p38 phosphorylation is necessary for the control of P2X7R expression, and activation of P2X7R would lead to the phosphorylation of p38. This is the first time to approach the question of the relationship between p38 phosphorylation and P2X7R expression in microglia of SDH.

### 3.7. IL-1*β*, IL-6, and TNF-*α* mRNA Expression in DRG and SDH after Inhibition of p38 Activation

**Figure 7:**
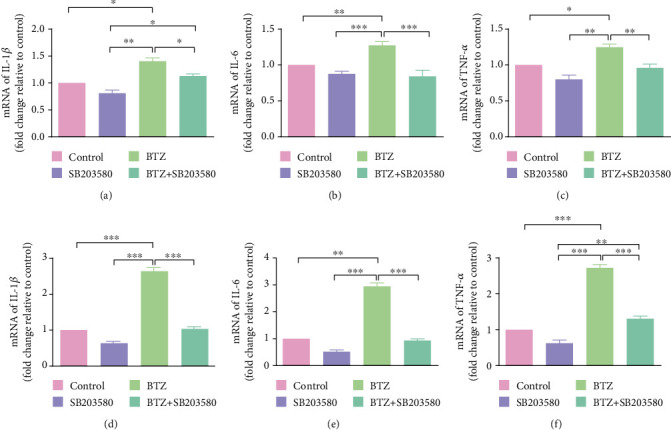
IL-1*β*, IL-6, and TNF-*α* mRNA expression in DRG and SDH after inhibition of p38 phosphorylation. (a) DRG IL-1*β* mRNA. (b) DRG IL-6 mRNA. (c) DRG TNF-*α* mRNA. (d) SDH IL-1*β* mRNA. (e) SDH IL-6 mRNA. (f) SDH TNF-*α* mRNA. Mean ± SEM (*n* = 5). ^∗^*P* < 0.05; ^∗∗^*P* < 0.01; ^∗∗∗^*P* < 0.001.

In this study, IL-1*β*, IL-6, and TNF-*α* mRNA expression levels were determined by using a quantitative real-time PCR technique in DRG and SDH from BTZ-treated rats with or without inhibition of p38 phosphorylation. The results showed that the elevated IL-1*β*, IL-6, and TNF-*α* mRNA levels in both DRG and SDH tissues induced by BTZ treatment could be significantly blocked by administration of the p38 selective inhibitor SB203580 (Figure 7). These results implied that BTZ-triggered inflammatory cytokine upregulation in both DRG and SDH tissues could be inhibited by inhibition of p38 activation, which is involved in mediating BTZ-induced peripheral neuropathy.

### 3.8. Mechanical Allodynia after Inhibition of P2X7R or p38

**Figure 8:**
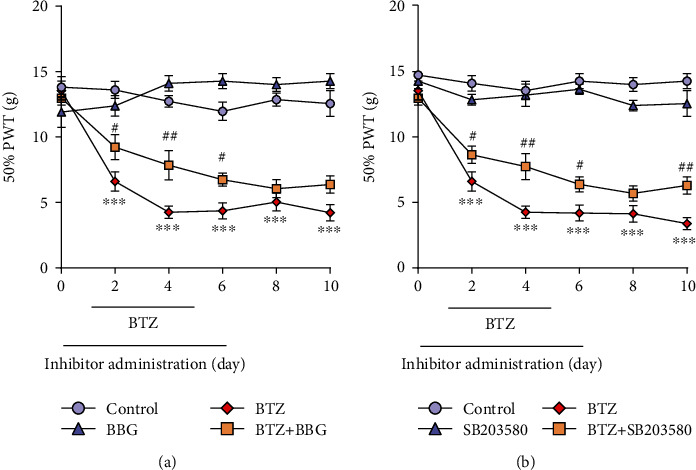
Mechanical threshold alterations after inhibition of P2X7R or p38. (a) Mechanical threshold after inhibition of P2X7R. (b) Mechanical threshold after inhibition of p38. Mean ± SEM (*n* = 5). ^∗∗∗^*P* < 0.001 (vs. control); ^#^*P* < 0.05; ^##^*P* < 0.01 (vs. BTZ group).

In the present study, a model of BTZ was used to stimulate a state of neuropathic pain. Applying behavioral measurements, we found that, compared with the BTZ group, intraperitoneal administration of the P2X7R selective inhibitor BBG before BTZ application could significantly improve the paw withdrawal thresholds for mechanical stimuli (Figure 8). Interestingly, BIPN rats showed a lower mechanical pain threshold, but the inhibitory effect disappeared at day 8. Compared with the control group, there was no statistical difference in the bilateral mechanical threshold in rats treated with BBG alone. This suggests that early intervention with BBG in BIPN rats could alleviate BTZ-induced pain. Lastly, we look insight into the relationship between BIPN and p38 activation. By blocking p38MAPK with SB203580, BIPN rats showed an identical performance of the BBG+BTZ group. The results of behavioral hypersensitivity assessment confirmed the activation of the P2X7R/p38MAPK signaling pathway after BIPN.

## 4. Discussion

Peripheral neuropathy is an obvious side effect of BTZ and a key factor leading to neurotoxicity. Although hyperalgesia was initially thought to be caused by changes in the activity of primary sensory neurons or neurons in the spinal cord, evidence has indicated that glial cells may also play a vital role in the development of pain. Nerve injury can cause spinal microglia P2X7R activation; as a result, the expression of inflammatory factors can be downregulated to reduce the pain-relevant behavioral hypersensitivity after P2X7R-specific inhibitor BBG interference [40]. Previous studies have confirmed that P2X7R activation in the DRG is involved in the communication between neurons and SGCs and plays a crucial role in mediating chronic and acute pain or nociception [16, 41]. In our present study, P2X7R was found in SGCs around DRG neurons by immunofluorescence labeling. BTZ administration induced the production of inflammatory cytokines, which play an important role in contributing to the pathogenesis and progression of neurotoxicity. We found that neuropathy caused by BTZ can activate P2X7R in DRG, and also confirmed that P2X7R is specifically expressed in the SGCs rather than in neurons, suggesting that glial cells in the peripheral nervous system indeed play a fundamental role in the process of BTZ-induced neuropathic pain.

The key step of p38MAPK phosphorylation in the central nervous system [7, 8] and peripheral nervous system [9–11] in mediating neuroinflammation and resulting in neuropathic pain has been approached recently in several studies. The importance of the involvement of p38MAPK phosphorylation in the development and maintenance of neuropathic pain boosts us to explore the association of p38MAPK phosphorylation and P2X7R activation in mediating BTZ-induced neuropathic pain. P2X7R has a crucial function in chronic pain or neuropathic pain in different pathological conditions, making P2X7R an appealing target for the treatment of intractable neuropathic pain [9, 17, 21, 42–44]. Exploring the expression and activation of P2X7R in DRG and SDH is particularly important for finding novel targets for relieving BTZ-induced neuropathic pain. In addition, BTZ-induced hypersensitivity and inflammatory factor release caused by activation of microglia in the SDH of rats are some of the key mechanisms leading to the development of neuropathic pain.

This study confirmed that BTZ can significantly activate P2X7R in SGCs of DRG, and P2X7R expression is consistent with the change of mechanical threshold in neuropathic pain rats.

Inhibition of P2X7R by intraperitoneal injection of BBG can result in downregulation of p-p38MAPK expression levels. Intrathecal administration of the p38MAPK inhibitor SB203580 significantly reduced the release of inflammatory factors, but had no effect on P2X7R expression. P2X7R association with p38MAPK in mediating inflammatory cytokine upregulation in DRG contributes to BTZ-induced mechanical allodynia represents a unique pharmacological property of P2X7R in mediating BTZ-induced neuropathic pain. Our results also showed that P2X7R is expressed specifically in microglial cells of SDH. Selective P2X7R antagonist BBG interference can obviously reduce the phosphorylation of p38MAPK in SDH. Inhibition of p38MAPK phosphorylation with SB203580 can result in downregulation of P2X7R expression levels in microglial cells of SDH. The association of P2X7R activation and p38MAPK phosphorylation in microglial cells of SDH also contributes to the development and progression of BTZ-induced neuropathic pain.

The evidence from clinical trial of patients with BTZ treatment [45] and experiment of BTZ-treated animals [46–48] supports that proinflammatory factors are closely correlated with the development of peripheral neuropathy or neuropathic pain. The application of the P2X7R inhibitor BBG prior to BTZ administration can significantly reduce TNF-*α*, IL-1*β*, and IL-6 mRNA expression in DRG and SDH as shown in these experiments. This result implied that P2X7R activity and p38MAPK phosphorylation may correlate with inflammatory factor production. It has been shown that enhanced expression of proinflammatory factors in DRG and signal transmission projects to the spinal cord seem to be associated with enhanced central hyperalgesia, which occurs after nerve injury or other neuropathic pain status [49, 50]. Therefore, further study on BTZ-induced central sensitization is worthy of exploration.

## 5. Conclusion

BTZ increased mechanical thresholds in rats, accompanied with upregulation of P2X7R expression and p38MAPK phosphorylation, indicating P2X7R and p38MAPK are key molecules in the development and maintenance of BTZ-induced neuropathic pain. Blocking p38MAPK phosphorylation with SB203580 can result in downregulating P2X7R expression levels. The application of P2X7R inhibitor BBG reversed neuropathic pain might decrease through the expression of TNF-*α*, IL-1*β*, and IL-6 via inhibiting p38MAPK phosphorylation. Exploring the association of P2X7R and the p38MAPK signaling pathway in mediating BTZ-induced neuropathic pain will allow a better understanding to pursue potential pharmacological targets behind this mechanism as an opportunity to relieve neuropathic pain.

## Figures and Tables

**Table 1:** The sequences of oligonucleotide primers.

Genes	Primer sequences
P2X7R	5′-CTACTCTTCGGTGGGGGCTT-3′ (coding sense)
	5′-CTCTGGATCCGGGTGACTTT-3′ (coding antisense)
IL-1*β*	5′-GGAAGGCAGTGTCACTCATTGTG-3′ (coding sense)
	5′-GGTCCTCATCCTGGAAGCTCC-3′ (coding antisense)
IL-6	5′-GGGACTGATGTTGTTGACAGCC-3′ (coding sense)
	5′-CATATGTAATTAAGCCTCCGACTTGTG-3′ (coding antisense)
TNF-*α*	5′'-CCCCGACTATGTGCTCCTCAC-3′ (coding sense)
	5′-AGGGCTCTTGATGGCGGA-3′ (coding antisense)
p38	5′-CTGCGAGGGCTGAAGTAT-3′ (coding sense)
	5′-TCCTCTTATCCGAGTCCAA-3′ (coding antisense)
GAPDH	5′-TCCCTCAAGATTGTCAGCAA-3′ (coding sense)
	5′-AGATCCACAACGGATACATT-3′ (coding antisense)

**Table 2:** The antibodies for immunoblotting.

Category	Antibodies	Concentration	Source
Primary	Rabbit anti-p-p38 monoclonal IgG	1 : 1000	Cell Signaling Technology, Danvers, MA
Primary	Rabbit anti-P2X7R monoclonal IgG	1 : 1000	Alomone Labs, Jerusalem, Israel
Primary	Rabbit anti-GAPDH monoclonal IgG	1 : 1000	Cell Signaling Technology, Danvers, MA
Primary	Mouse anti-*β*-actin monoclonal IgG	1 : 5000	Cell Signaling Technology, Danvers, MA
Secondary	Goat anti-rabbit IgG-HRP	1 : 5000	Beijing Sequoia Jinqiao Biological Technology Co., Ltd., Beijing, China
Secondary	Goat anti-mouse IgG-HRP	1 : 5000	Beijing Sequoia Jinqiao Biological Technology Co., Ltd., Beijing, China

**Table 3:** The antibodies for fluorescence labeling.

Category	Antibodies	Concentration	Source
Primary	Rabbit anti-P2X7R monoclonal IgG	1 : 400	Alomone Labs, Jerusalem, Israel
Primary	Rabbit anti-p-p38 monoclonal IgG	1 : 100	Cell Signaling Technology, Danvers, MA
Primary	Chicken anti-NF-200 monoclonal IgG	1 : 1000	Cell Signaling Technology, Danvers, MA
Primary	Mouse anti-F4/80 monoclonal IgG	1 : 100	Santa Cruz Biotechnology, Santa Cruz, CA
Primary	Mouse anti-Iba-1 monoclonal IgG	1 : 200	Abcam, Cambridge, MA
Primary	Mouse anti-GFAP monoclonal IgG	1 : 500	Abcam, Cambridge, MA
Secondary	Goat anti-rabbit IgG-TRITC	1 : 200	Beijing Sequoia Jinqiao Biological Technology Co., Ltd., Beijing, China
Secondary	Goat anti-chicken IgG-FITC	1 : 200	Beijing Sequoia Jinqiao Biological Technology Co., Ltd., Beijing, China
Secondary	Goat anti-mouse IgG-TRITC	1 : 200	Beijing Sequoia Jinqiao Biological Technology Co., Ltd., Beijing, China
Secondary	Goat anti-mouse IgG-FITC	1 : 500	Beijing Sequoia Jinqiao Biological Technology Co., Ltd., Beijing, China

## Data Availability

The data used to support the findings in this study are available from the corresponding author upon reasonable request.
